# Editorial Promoting Help-seeking using E-Technology for ADolescents: The ProHEAD consortium

**DOI:** 10.1186/s13063-018-3162-x

**Published:** 2019-01-22

**Authors:** Michael Kaess, Stephanie Bauer

**Affiliations:** 1 Section for Translational Psychobiology in Child and Adolescent Psychiatry, Department of Child and Adolescent Psychiatry, University Hospital Heidelberg, Blumenstraße 8, 69115 Heidelberg, Germany; 2 University Hospital of Child and Adolescent Psychiatry and Psychotherapy, University of Bern, Bolligenstr. 111, Stöckli, 3000 Bern, Switzerland; 3 Center for Psychotherapy Research, University Hospital Heidelberg, Bergheimerstr. 54, 69115 Heidelberg, Germany

## Abstract

Mental health problems are highly prevalent in children and adolescents, but professional help-seeking behavior in this age group is extremely low. Therefore, the ProHEAD (“Promoting Help-seeking using E-technology for Adolescents”) consortium focuses on three main objectives, i.e.: (1) improving young people’s help-seeking behaviors; (2) improving the selective prevention of common disorders in those who are at risk; and (3) strengthening resources to counteract the development of mental illness. Capitalizing on Internet and mobile technology, ProHEAD delivers low threshold and easily accessible interventions to a large sample of young people. Longitudinal school-based assessments of mental health problems will be conducted at baseline and two annual follow-ups in five regions of Germany in a total sample of 15,000 children and adolescents (aged ≥ 12 years). Based on the results of their baseline assessment, participants are invited to register for one out of five sub-projects. The objectives and procedures of these five randomized controlled trials are published in this issue of *Trials*.

## Introduction

The individual, societal, and economical burden of mental illness is huge. Almost half of the entire population will be affected by at least one form of mental illness at some point during their lifetime; for many individuals, illness onset occurs during childhood or adolescence. About three-fourths of all lifetime cases develop before the age of 25 years; for some diagnoses such as anxiety or impulse-control disorder, the median age of onset is as early as 11 years [1]. Poor mental health in childhood and adolescence may have lifelong consequences. It is associated with an increased risk of mental illness in adulthood as well as with an increased risk for impaired academic and professional achievements and overall reduced quality of life [2]. These facts clearly illustrate the need for prevention, early identification, and timely treatment of mental health problems.

The growing literature on evidence-based care for mental illness in children and adolescents convincingly documents that professional treatment leads to lasting benefits and full recovery in many youth (e.g. [3, 4]). Early intervention may improve the long-term prognosis and reduce the risk of chronic courses of illness (e.g. [5]).

However, many children and adolescents never access specialist services for their mental health problems or do so only long after the onset of initial symptoms. This so-called “treatment gap” and the resulting unmet needs concerning the mental health of young people have been identified for various diagnoses and for different countries and healthcare settings [6–8]. A number of factors contribute to the reluctance to seek professional help. These include stigma, embarrassment, poor mental health literacy (i.e. poor ability to recognize symptoms and poor knowledge about mental health treatment), and a preference for self-reliance [9]. In addition, factors such as poor parental understanding of both mental illness and the help-seeking process as well as negative parental attitudes towards treatment seem to play a role [10]. Only few studies have investigated factors that are associated with an increased likelihood of help-seeking in young people. Such facilitators are positive experiences with professional support in the past, social support, and encouragement from others [9].

Low rates of help-seeking are not only problematic on an individual level but also on a societal, economic, and public health level. From a public health perspective, the reach of an intervention is equally important as its effectiveness [11]. This means that even highly effective treatment programs may result in a very small reduction of the burden of illness on a population level if they are utilized only by a small proportion of the target population. Improving young people’s help-seeking behaviors would enhance the reach of evidence-based care and is thus considered a priority in health service research. Other key factors in order to limit the burden associated with mental illness include enhancing the reach and effectiveness of preventive efforts and health promotion programs, which would result in reduced rates of illness and higher proportions of young people staying healthy throughout childhood and adolescence.

Thus, the ProHEAD consortium which is introduced in the present issue of *Trials* addresses three overall objectives, i.e.: (1) improving young people’s help-seeking behaviors and thus increasing rates of treatment uptake in those who experience clinically relevant mental health problems; (2) improving the selective prevention of common disorders in young people by reducing symptoms of depression, eating disorders, and substance abuse in those who are at risk; and, finally, (3) strengthening skills and resources to avoid the development of mental illness in those who are currently healthy.

This collaborative effort to enhance care for young people across the service spectrum from health promotion to prevention to treatment requires a comprehensive approach that allows us: (1) to screen for mental health problems in large samples of children and adolescents and to give timely feedback on the results of the assessment; (2) to provide tailored interventions depending on the individual type and level of impairment; and (3) to minimize psychological barriers (e.g. uncertainties, concerns related to confidentiality, shame, fear of stigmatization) as well as practical barriers such as waiting times, travel times, and office hours that may prevent young people from accessing services. Therefore, ProHEAD capitalizes on Internet and mobile technology to deliver easily accessible, permanently available, and trustworthy tools specifically developed to meet the needs and communication preferences of young people.

The vast majority of today’s youth have access to the Internet, most own a Smartphone, and many look up information about mental health problems and their treatment online [12]. Without any doubt, online and mobile services will continue to become more and more integrated into all areas of young people’s lives including medical and therapeutic services. The past two decades have already shown a rapidly increasing number of technology-enhanced interventions for the prevention, self-help, and treatment of various types of mental illness in adults [13–15] as well as in children and adolescents [16, 17]. However, only few studies addressed the question to which extent online interventions may facilitate offline help-seeking of conventional mental healthcare [18]. While there is overall broad consensus on the acceptability and positive effects that technology-enhanced delivery of care may have in specific settings, there are also some important gaps in the literature. One of the major limitations of past research concerns the fact that the majority of studies includes self-selected samples of individuals that may already have a preference for online services. To our knowledge, ProHEAD is the first consortium that uses a school-based approach in order to screen for a broad range of mental health problems among a large sample of children and adolescents, and at the same time invites each participant to register for one out of several online interventions depending on their individual symptom profile. This procedure promises to significantly advance our knowledge on the reach, uptake, efficacy, and cost-effectiveness of technology-enhanced programs for the promotion of mental health, the prevention of mental illness, and the facilitation of access to professional mental healthcare.

## The ProHEAD consortium

**Fig. 1:**
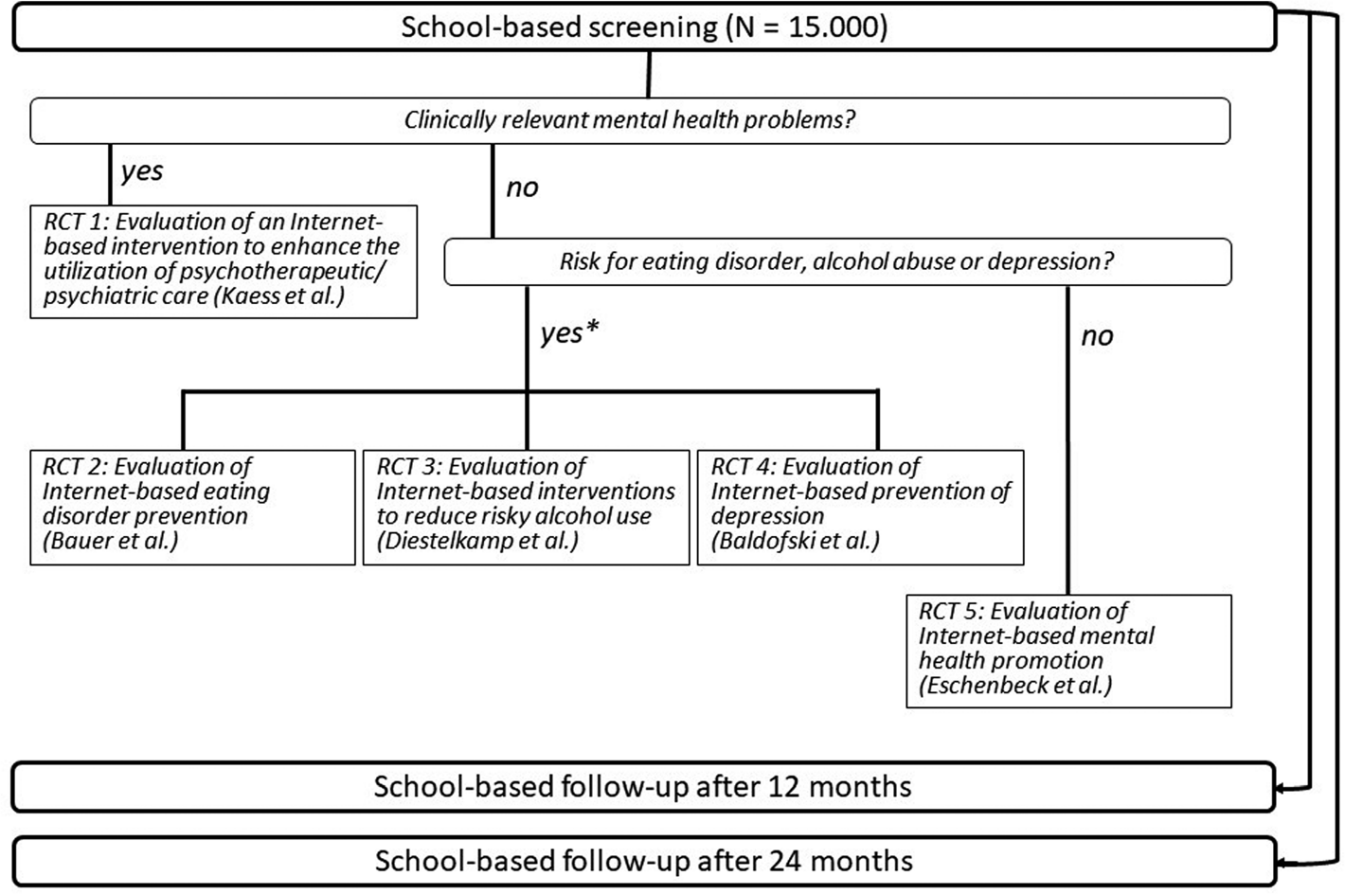
Overview of the five RCTs/sub-projects within ProHEAD, Note: * Participants who meet eligibility criteria for more than one RCT are randomly allocated to one of the three sub-projects

The ProHEAD consortium will conduct longitudinal school-based assessments of mental health problems in a sample of 15,000 children and adolescents aged ≥ 12 years. Online assessments including an initial baseline screening and two annual follow-ups will be conducted at a randomly selected sample of high schools in five regions of Germany. Following the completion of the screening assessment, participants receive feedback on their individual results along with an invitation to register for one out of five sub-projects (Fig. 1). These focus on the improvement of help-seeking behaviors in participants reporting a clinically relevant level of impairment (sub-project 1; (Kaess M, Ritter S, Lustig S, Bauer S, Becker K, Eschenbeck H, et al.: Promoting Help-seeking using E-technology for Adolescents with Mental Health Problems: Study Protocol for a Randomized Controlled Trial within the ProHEAD Consortium. Trials, submitted), the selective prevention of eating disorders (sub-project 2; (Bauer S, Bilić S, Reetz C, Ozer F, Becker K, Eschenbeck H, et al.: Efficacy and Cost-Effectiveness of Internet-based Selective Eating Disorder Prevention: Study Protocol for a Randomized Controlled Trial within the ProHEAD Consortium. submitted), alcohol abuse (sub-project 3; (Diestelkamp S, Wartberg L, Kaess M, Bauer S, Rummel-Kluge C, Becker K, et al.: Effectiveness of a web-based screening and brief intervention with weekly text-message-initiated individualised prompts for reducing risky alcohol use among teenagers: study protocol of a randomised controlled trial within the ProHEAD Consortium, submitted) and depression (sub-project 4; (Baldofski S, Kohls E, Bauer S, Becker K, Bilic S, Eschenbeck H, et al.: Efficacy and Cost-Effectiveness of Two Online Interventions for Children and Adolescents at Risk for Depression (E.motion trial): Study Protocol for a Randomized Controlled Trial within the ProHEAD Consortium, submitted) in participants at risk for these conditions, and finally on the promotion of mental health in participants who do not report an increased level of risk or impairment in the baseline screening (sub-project 5; (Eschenbeck H, Lehner L, Hofmann H, Bauer S, Becker K, Diestelkamp S, et al.: School-Based Mental Health Promotion in Children and Adolescents with StresSOS using Online or Face-to-Face Interventions: Study Protocol for a Randomized Controlled Trial within the ProHEAD Consortium, submitted).

The study protocols published in this issue of *Trials* provide a detailed overview on the objectives and procedures of the five randomized controlled trials (RCTs) that will be conducted as sub-projects within ProHEAD. It should be noted that all five RCTs include active control groups, i.e. each participating student will be invited to engage in an intervention following the initial screening.

In sub-project 1, Kaess et al. (Kaess M, Ritter S, Lustig S, Bauer S, Becker K, Eschenbeck H, et al.: Promoting Help-seeking using E-technology for Adolescents with Mental Health Problems: Study Protocol for a Randomized Controlled Trial within the ProHEAD Consortium. Trials, submitted) will investigate the efficacy and cost-effectiveness of a new online intervention (“ProHEAD online”) developed to facilitate access to conventional mental healthcare. Participants will have access to tailored information, case reports, peer support, and individual counseling sessions with a clinician (online and via phone) on their need for professional help as well as ways to overcome potential barriers that may impede help-seeking in their individual case. Primary outcome will be the rate of participants who actually utilize professional mental healthcare within one year after the initial screening.

In sub-project 2, Bauer et al. (Bauer S, Bilić S, Reetz C, Ozer F, Becker K, Eschenbeck H, et al.: Efficacy and Cost-Effectiveness of Internet-based Selective Eating Disorder Prevention: Study Protocol for a Randomized Controlled Trial within the ProHEAD Consortium, submitted) will study the efficacy and cost-effectiveness of two new versions of an existing intervention for the prevention of eating disorders (“ProYouth”). Based on latest evidence, one of the enhanced versions of the program includes a dissonance-based module encouraging participants to challenge the current beauty ideals related to male and female bodies. The other version includes a structured module based on the principles of cognitive behavioral therapy addressing body image concerns. It is assumed that both interventions prove superior compared to ProYouth alone in terms of a reduction of eating disorder related impairment.

Sub-project 3 focuses on the potential of a single session brief motivational web-based intervention (“Pro-WISE”) plus weekly text-message-initiated individualized prompts (TIPs) in participants who screen positive for harmful alcohol use. Diestelkamp et al. (Diestelkamp S, Wartberg L, Kaess M, Bauer S, Rummel-Kluge C, Becker K, et al.: Effectiveness of a web-based screening and brief intervention with weekly text-message-initiated individualised prompts for reducing risky alcohol use among teenagers: study protocol of a randomised controlled trial within the ProHEAD Consortium, submitted) hypothesize that the TIPs will result in reduced alcohol consumption compared to the control conditions because they may reach participants in the contexts of their everyday lives by providing individualized feedback on drinking intentions, actual drinking, and the ability to achieve personal goals for low-risk drinking or abstinence.

In sub-project 4, Baldofski et al. (Baldofski S, Kohls E, Bauer S, Becker K, Bilic S, Eschenbeck H, et al.: Efficacy and Cost-Effectiveness of Two Online Interventions for Children and Adolescents at Risk for Depression (E.motion trial): Study Protocol for a Randomized Controlled Trial within the ProHEAD Consortium, submitted) will investigate the efficacy and cost-effectiveness of online support for children and adolescents at risk for depression. Two interventions (i.e. the guided self-help program “iFightDepression” and a clinician-guided group chat intervention) will be compared to a control condition (i.e. online psychoeducational content) in terms of the reduction of depressive symptomatology.

Finally, in sub-project 5, Eschenbeck et al. (Eschenbeck H, Lehner L, Hofmann H, Bauer S, Becker K, Diestelkamp S, et al.: School-Based Mental Health Promotion in Children and Adolescents with StresSOS using Online or Face-to-Face Interventions: Study Protocol for a Randomized Controlled Trial within the ProHEAD Consortium, submitted) will evaluate a newly developed online version of a universal school-based health promotion program (“StresSOS”) aiming to enhance stress management skills and mental health literacy in participants who do not report relevant mental health problems in the baseline screening. The online version of StresSOS will be compared to an active online control condition as well as to the original version of the intervention delivered in the face-to-face setting. The primary outcome will be the rate of participants who are classified as high-risk or clinically relevant impaired at 12-month follow-up.

Overall, the ProHEAD consortium has been established to address the urgent need for innovative and widely accessible initiatives for the prevention as well as early identification and timely intervention in child and adolescent mental health. Facts such as the high incidence rates of mental illness in childhood and adolescence, low rates of seeking professional help, as well as delayed identification of mental health problems and treatment uptake are alarming and challenging. The young age is a critical period to implement interventions and the school setting appears to be an ideal environment to approach children and adolescents. Within ProHEAD, only the annual assessments will actually be conducted at the schools and all interventions will be delivered electronically outside of the school setting. This procedure of combining the school-based assessments and the technology-enhanced interventions has several advantages. First, it allows us to invite a representative sample of students to participate in the project independent of their previous knowledge or attitudes towards mental illness or e-health as well as independent of their perceived need for support. The results concerning the uptake and utilization of the various online interventions will draw a realistic picture of young people’s interest and willingness to engage in such tools. Second, the school-based approach will ensure high data quality due to the expected high completion rates of school-based follow-up assessments. Third, the use of modern technology-enhanced tools such as the ProHEAD online platform does not only maximize our chances to reach large samples of young people but may also lay the foundation for the future large-scale dissemination of successfully evaluated interventions. The long-term sustainability of the interventions studied as part of ProHEAD does of course not only depend on their efficacy and effectiveness, but ultimately also on their costs and health-economic evaluation. Therefore, all sub-projects within ProHEAD include cost-effectiveness and cost-utility analyses in order to allow for informed decision-making following the end of the study period.

## References

[1] Kessler RC, Berglund P, Demler O, Jin R, Merikangas KR, Walters EE (2005). Lifetime prevalence and age-of-onset distributions of DSM-IV disorders in the National Comorbidity Survey Replication. Arch Gen Psychiatry.

[2] Patton GC, Coffey C, Romaniuk H, Mackinnon A, Carlin JB, Degenhardt L (2014). The prognosis of common mental disorders in adolescents: a 14-year prospective cohort study. Lancet.

[3] Gálvez-Lara M, Corpas J, Moreno E, Venceslá JF, Sánchez-Raya A, Moriana JA (2018). Psychological treatments for mental disorders in children and adolescents: a review of the evidence of leading international organizations. Clin Child Fam Psychol Rev.

[4] Pu J, Zhou X, Liu L, Zhang Y, Yang L, Yuan S (2017). Efficacy and acceptability of interpersonal psychotherapy for depression in adolescents: A meta-analysis of randomized controlled trials. Psychiatry Res.

[5] Patel V, Flisher AJ, Hetrick S, McGorry P (2007). Mental health of young people: a global public-health challenge. Lancet.

[6] Essau CA (2005). Frequency and patterns of mental health services utilization among adolescents with anxiety and depressive disorders. Depress Anxiety.

[7] Merikangas KR, He JP, Burstein M, Swendsen J, Avenevoli S, Case B (2011). Service utilization for lifetime mental disorders in US adolescents: results of the National Comorbidity Survey–Adolescent Supplement (NCS-A). J Am Acad Child Adolesc Psychiatry.

[8] Xu Z, Huang F, Kösters M, Staiger T, Becker T, Thornicroft G (2018). Effectiveness of interventions to promote help-seeking for mental health problems: systematic review and meta-analysis. Psychol Med.

[9] Gulliver A, Griffiths KM, Christensen H (2010). Perceived barriers and facilitators to mental health help-seeking in young people: a systematic review. BMC Psychiatry.

[10] Reardon T, Harvey K, Baranowska M, O’Brien D, Smith L, Creswell C (2017). What do parents perceive are the barriers and facilitators to accessing psychological treatment for mental health problems in children and adolescents? A systematic review of qualitative and quantitative studies. Eur Child Adolesc Psychiatry.

[11] Glasgow RE, Lichtenstein E, Marcus AC (2003). Why don’t we see more translation of health promotion research to practice? Rethinking the efficacy-to-effectiveness transition. Am J Public Health.

[12] Mitchell C, McMillan B, Hagan T (2017). Mental health help-seeking behaviours in young adults. Br J Gen Pract.

[13] Carlbring P, Andersson G, Cuijpers P, Riper H, Hedman-Lagerlöf E (2018). Internet-based vs. face-to-face cognitive behavior therapy for psychiatric and somatic disorders: an updated systematic review and meta-analysis. Cogn Behav Ther.

[14] Kampmann IL, Emmelkamp PM, Morina N (2016). Meta-analysis of technology-assisted interventions for social anxiety disorder. J Anxiety Disord.

[15] Karyotaki E, Riper H, Twisk J, Hoogendoorn A, Kleiboer A, Mira A (2017). Efficacy of self-guided internet-based cognitive behavioral therapy in the treatment of depressive symptoms: a meta-analysis of individual participant data. JAMA Psychiatry.

[16] Ebert DD, Zarski AC, Christensen H, Stikkelbroek Y, Cuijpers P, Berking M (2015). Internet and computer-based cognitive behavioral therapy for anxiety and depression in youth: a meta-analysis of randomized controlled outcome trials. PLoS One.

[17] Vigerland S, Lenhard F, Bonnert M, Lalouni M, Hedman E, Ahlen J (2016). Internet-delivered cognitive behavior therapy for children and adolescents: a systematic review and meta-analysis. Clin Psychol Rev.

[18] Kauer SD, Mangan C, Sanci L (2014). Do online mental health services improve help-seeking for young people? A systematic review. J Med Internet Res.

